# DMSO-mediated curing of several yeast prion variants involves Hsp104 expression and protein solubilization, and is decreased in several autophagy related gene (*atg*) mutants

**DOI:** 10.1371/journal.pone.0229796

**Published:** 2020-03-05

**Authors:** Jane E. Dorweiler, Joanna O. Obaoye, Mitch J. Oddo, Francesca M. Shilati, Grace M. Scheidemantle, Thomas J. Coleman, Jacob A. Reilly, Gregory R. Smith, Anita L. Manogaran

**Affiliations:** 1 Department of Biological Sciences, Marquette University, Milwaukee, WI, United States of America; 2 Department of Biology, Lakeland University, Plymouth, WI, United States of America; University of Pittsburgh, UNITED STATES

## Abstract

Chaperones and autophagy are components of the protein quality control system that contribute to the management of proteins that are misfolded and aggregated. Here, we use yeast prions, which are self-perpetuating aggregating proteins, as a means to understand how these protein quality control systems influence aggregate loss. Chaperones, such as Hsp104, fragment prion aggregates to generate more prion seeds for propagation. While much is known about the role of chaperones, little is known about how other quality control systems contribute to prion propagation. We show that the aprotic solvent dimethyl sulfoxide (DMSO) cures a range of [*PSI*^+^] prion variants, which are related to several misfolded aggregated conformations of the Sup35 protein. Our studies show that DMSO-mediated curing is quicker and more efficient than guanidine hydrochloride, a prion curing agent that inactivates the Hsp104 chaperone. Instead, DMSO appears to induce Hsp104 expression. Using the yTRAP system, a recently developed transcriptional reporting system for tracking protein solubility, we found that DMSO also rapidly induces the accumulation of soluble Sup35 protein, suggesting a potential link between Hsp104 expression and disassembly of Sup35 from the prion aggregate. However, DMSO-mediated curing appears to also be associated with other quality control systems. While the induction of autophagy alone does not lead to curing, we found that DMSO-mediated curing is dramatically impaired in autophagy related (*atg*) gene mutants, suggesting that other factors influence this DMSO mechanism of curing. Our data suggest that DMSO-mediated curing is not simply dependent upon Hsp104 overexpression alone, but may further depend upon other aspects of proteostasis.

## Introduction

Protein quality control mechanisms, which include molecular chaperones, autophagy, and the ubiquitin proteasome system, ensure that misfolded proteins are either refolded or targeted for degradation. However, aging and environmental stress can impact the balance of these protein quality control pathways, resulting in large impacts on protein homeostasis. In addition, several human diseases associated with protein aggregation such as prion disease, Alzheimer’s disease and amyotrophic lateral sclerosis (ALS) are linked with decline in protein quality control activity [reviewed in 1]. Understanding how activation of protein quality control pathways can alleviate detrimental impacts associated with age, stress, or disease provides important directions for therapeutic targets.

The study of prions, or misfolded infectious proteins, in *Saccharomyces cerevisiae* has facilitated our understanding of how protein quality control pathways help manage protein aggregates. The [*PSI*^+^] prion is the misfolded aggregated version of the Sup35 protein, which is normally involved in translation termination [reviewed in 2]. Work with [*PSI*^+^] has established that molecular chaperones play an important role in propagating prions within cell populations. Hsp104, an AAA+ ATPase, along with Hsp40 and Hsp70 family chaperones, acts as a general protein disaggregase [3]. In the case of prions, Hsp104 is able to fragment prion aggregates into two by extracting a monomer from within the aggregate. This process generates more prion seeds, which is necessary for propagation [4–10]. Alterations in chaperone levels have been shown to impact the ability of prions to propagate [4]. Growth of [*PSI*^+^] cells in the presence of low concentrations of guanidine hydrochloride (GuHCl) leads to functional Hsp104 inactivation, resulting in prion loss, also known as curing [10, 11]. Prion loss through GuHCl treatment arrests prion replication. As a result, the remaining propagons are diluted over multiple cell divisions [4, 6, 7, 12, 13].

Insight into the dynamics that contribute to prion loss has been facilitated through the study of prion variants. The [*PSI*^+^] prion can exist in a wide variety conformations [14–16]. These variants are associated with different amounts of aggregated protein, distinct biochemical characteristics, and specific phenotypes [7, 14–18]. Two examples of prion variants are the weak [*PSI*^+^] and the strong [*PSI*^+^] variant [14]. Weak [*PSI*^+^] is more unstable, has larger propagon sizes, and has a larger pool of Sup35 monomers, compared to strong [*PSI*^+^]. Transient heat shock has been shown to cause the curing of weak, but not strong, [*PSI*^+^] variants [19]. It has been recently shown that during transient heat shock, the retention of Hsp104 in mother cells is correlated with loss of the prion from the population suggesting that spatial sequestration of Hsp104 mediates prion curing [20, 21].

The role of chaperones in prion propagation cannot be disputed; however, there is little known regarding how alterations of other protein quality control pathways can lead to prion curing. Here, we use the aprotic solvent, dimethyl sulfoxide (DMSO) to investigate other mechanisms of prion curing. It was previously shown that DMSO treatment can lead to [*PSI*^+^] loss [22, 23]. Here, we find DMSO causes the rapid loss of many [*PSI*^+^] variants, including weak [*PSI*^+^]. DMSO-mediated curing is associated with enhanced Hsp104 expression, and is dampened in autophagy related gene mutants. Our studies suggest that DMSO-mediated [*PSI*^+^] curing involves chaperones and processes that may be linked to autophagy related genes.

## Results

### DMSO cures several prion variants, but not strong *[PSI*^*+*^*]*

**Fig 1:**
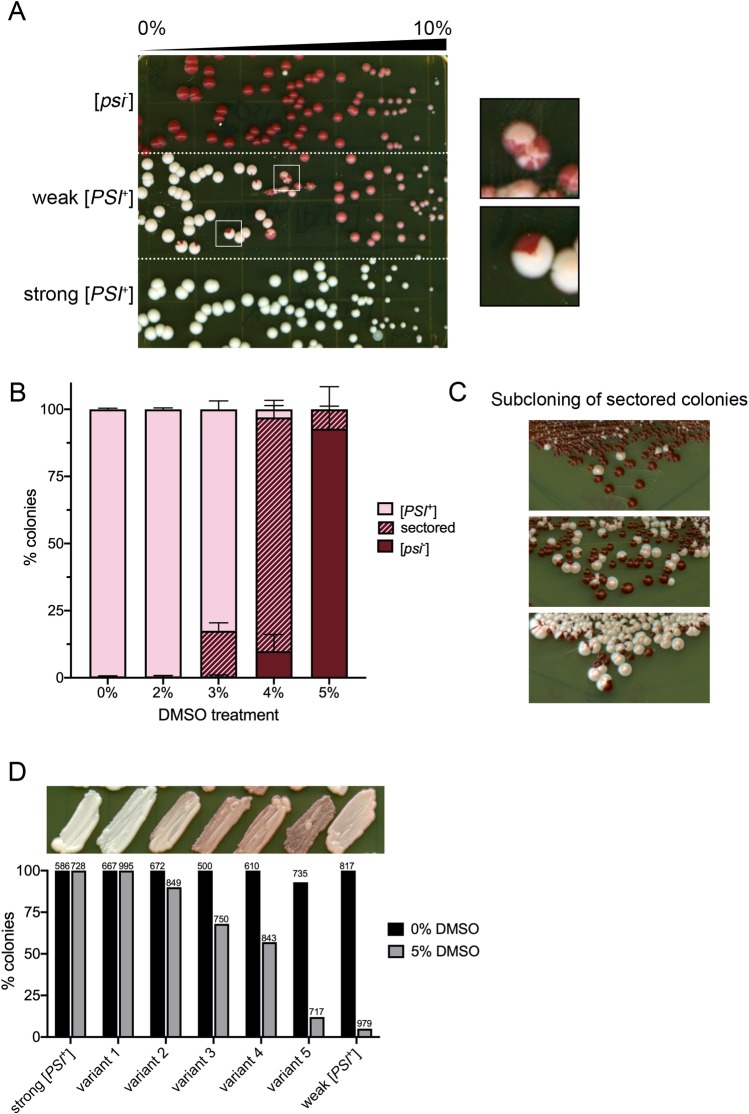
Many *[PSI*^*+*^*]* variants are cured by DMSO treatment. A. The indicated strains were plated on 0–10% DMSO gradient plates, and assayed for colony color after growth. Inset shows sectored colonies observed in weak [*PSI*^+^] strains. Plate is representative of several trials by independent researchers. B. Weak [*PSI*^+^] strains were grown overnight and plated onto rich media plates containing the indicated concentration of DMSO. Each bar shows triplicate trials representing a total of 700 colonies or more. C. Sectored colonies from 4% DMSO were re-streaked on rich media, and assayed for colony color after growth. Images are representative of streaks obtained. D. [*PSI*^+^] variants were induced *de novo*, and a range of color variants were chosen for analysis (variant 1–5; top panel). It should be noted that variant 5 was slightly unstable when plated on rich media. Each variant was cultured in the absence (0%) or presence (5%) of DMSO overnight plated on rich media, and assessed for colony color. The number of colonies counted per treatment is shown.

The presence of [*PSI*^+^] can be easily monitored using a colony color assay in strains carrying an *ade1-14* nonsense allele [4]. On rich media, different variants of [*PSI*^+^] have different colony color phenotypes. [*psi*^*-*^] strains produce red colonies, which is the *ade1-14* phenotype. [*PSI*^+^] variants with low levels of soluble Sup35 allow nonsense read through, suppressing the *ade1-14* phenotype, producing white colonies. In contrast, [*PSI*^+^] variants with more soluble Sup35 have less read through, producing pink colonies [Fig 1A; 14, 17, 18, 24].

We were interested in how DMSO mediates prion curing. To start, we tested two established variants of [*PSI*^+^], weak [*PSI*^+^] and strong [*PSI*^+^] [14]. [*psi*^-^], weak [*PSI*^+^] and strong [*PSI*^+^] cells were plated on gradient plates, where DMSO was present from 0–10%. As previously reported [22], all strains displayed toxicity at concentrations nearing 10% DMSO. While DMSO treatment did not impact colony color of [*psi*^-^] or strong [*PSI*^+^] strains, weak [*PSI*^+^] colonies exhibited a change of color from pink to red within the gradient (Fig 1A). Interestingly, weak [*PSI*^+^] cells plated on gradient plates gave rise to sectored colonies at concentrations roughly equivalent to 2–5% DMSO (Fig 1A, inset). These sectored colonies suggest that the cell that was originally plated was phenotypically [*PSI*^+^], but during cell division, one or more of the progeny lost the prion. Subsequent divisions of these cured progeny gave rise to the red sector.

Next, we plated weak [*PSI*^+^] on plates containing finite concentrations of DMSO. This approach allows us to quantify curing in a large population of weak [*PSI*^*+*^] cells, compared to the qualitative analysis of gradients plates. We plated multiple independent weak [*PSI*^+^] cultures on 0%, 2%, 3%, 4% and 5% DMSO and allowed cells to form colonies. Almost all the colonies treated with 2% DMSO retained their weak [*PSI*^+^] status, but noticeable differences were observed with higher concentrations. 3 and 4% DMSO treatment gave rise on average to 16.9% and 87.0% sectored colonies, respectively (Fig 1B). Sectored colonies were further subcloned and gave rise to only red or pink colonies, indicating that sectored colonies are comprised of cells that are either [*PSI*^+^] or [*psi*^-^] (Fig 1C). 5% DMSO treatment gave rise to an average of 92.5% red colonies, which are [*psi*^-^]. Again, further subcloning of red colonies resulted in red colonies (S1 Fig). However, it should be noted that even colonies that appeared to be phenotypically [*PSI*^+^] after DMSO treatment were often unstable upon restreaking, resulting in either red or white colonies (S1 Fig). Our data indicate that DMSO treatment leads to the irreversible loss of weak [*PSI*^+^].

To determine whether DMSO could cure other variants, we induced the formation of several new [*PSI*^+^] variants *de novo* using previously described methods [25, 26]. The color of *de novo* variants obtained ranged from white to dark pink. Most strains maintained colony color after subsequent restreaking on rich media, but similar to previous reports some variants were unstable [17, 27]. Several *de novo* variants were chosen with a wide range of colony color phenotypes (Fig 1D, top panel), including one unstable variant (variant 5). The degree of curing by 5% DMSO appeared to be directly related to the colony color phenotype of the *de novo* obtained variants. Strains that had a darker colony color were more prone to DMSO curing than lighter ones. It should be noted that white strain (variant 1) was highly resistant DMSO curing, similar to strong [*PSI*^+^]. However weak [*PSI*^+^], which had a lighter colony color than some of our *de novo* obtained variants, had the greatest overall curing (Fig 1D).

### DMSO-mediated curing is faster than GuHCl curing

**Fig 2:**
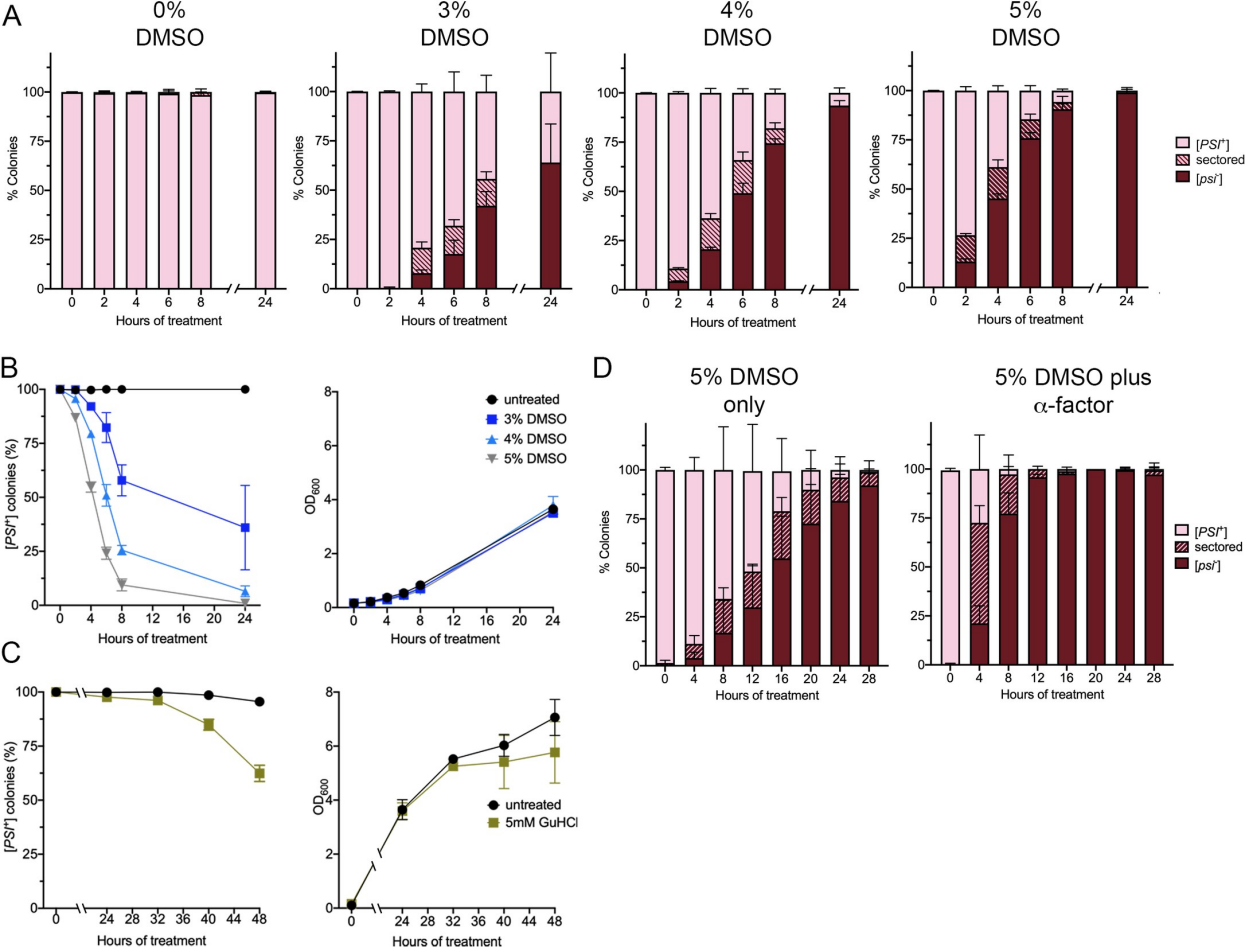
DMSO curing occurs quickly and is enhanced by α-factor treatment. A. Three separate weak [*PSI*^+^] cultures were grown overnight, and then inoculated into media that contained either 3%, 4%, or 5% DMSO, or no DMSO (0%), as indicated. Cultures were inoculated with starting optical densities of 0.156. At specific time points, cultures were plated on rich media and colonies were assessed for colony color. Each bar represents approximately 100 colonies per trial (in triplicate) were scored per time points. Data represents colonies that are [*psi*^*-*^] (red), sectored (striped), and [*PSI*^+^] (pink). B. Datasets from A were plotted to observe the rate of [*PSI*^+^] loss over time (left panel). Culture growth, monitored by optical density, is shown (right panel). C. Untreated (black), or cultures treated with 5mM GuHCl were inoculated to an OD_600_ of 0.1. Similar to part B, colonies were scored for [*PSI*^+^] over time (left panel) and cultures were tested for optical density (right panel). A minimum of 380 colonies per trial (in triplicate) were scored per time point. Note that the time scale is different on the x-axis between B and C. D. Three separate weak [*PSI*^+^] cultures were grown overnight, and then inoculated into media that contained either 5% DMSO, or 5% DMSO with 50 μM α-factor, as indicated. Cultures were inoculated with starting optical densities of approximately 0.01. Each bar represents approximately 200 colonies in triplicate. All data represents means; error bars represent standard deviations (n = 3 or more).

Transient heat treatment of weak [*PSI*^+^] cells causes quick prion loss by inducing Hsp104 expression and retaining Hsp104 in mother cells [19–21]. In contrast, low level of GuHCl treatment mediates slow prion loss through Hsp104 inactivation. Inactivation is associated with the gradual dilution of propagons with cell division, resulting in curing occurring after many cell doublings [11, 13]. Therefore, the rate of curing by DMSO could provide insight into how the prion is lost. Cells were inoculated in liquid culture at an optical density of 0.15, supplemented with different concentrations of DMSO, and allowed to grow. At different time points, cultures were plated on rich media to assess the percentage of colonies that were cured. Cultures treated with 3%, 4%, and 5% DMSO all exhibited some prion loss as quickly as four hours of treatment (Fig 2A and 2B). The most dramatic effect of curing was observed with 5% DMSO. 38.8% of the plated colonies retained [*PSI*^+^] after four hours of treatment and almost all the colonies were [*psi*-] by eight hours of treatment. Only 5.73% of the colonies were [*PSI*^+^]. In contrast, no GuHCl-mediated curing of [*PSI+*] was not observed between four and 24 hours. The appearance of [*psi-*] colonies was first observed at 32 hours of treatment, and 62.4% of the plated colonies retained [*PSI*^+^] after 48 hours of treatment (Fig 2C). The slope of curing observed in DMSO cultures is much steeper than that observed by 5mM GuHCl cultures (Fig 2B and 2C), suggesting that the mode of curing between the two treatments are different.

The quick curing by DMSO in liquid culture suggested that cell division in the presence of DMSO may contribute to curing. Therefore, we tested whether arresting the cells would impact DMSO-mediated curing. α -factor is a mating hormone that arrests cells in G1 of the cell cycle. To test whether cell cycle arrest would influence curing, weak [*PSI*^+^] cells were inoculated in liquid culture supplemented with 5% DMSO or 5% DMSO with α -factor at an optical density of approximately 0.015. After specific times of incubations, optical densities were measured to confirm that α -factor treated cells underwent cell cycle arrest (S2A Fig), and cultures were plated and scored for [*PSI*^+^]. Despite being completed by independent investigators and different starting optical density, 5% DMSO treated cultures (no α -factor) showed curing (Fig 2A and 2D). With 5% DMSO only, the number of sectored colonies maintained within the population between 4 and 20 hours was between 7–24% (Fig 2D), and only 65.8% of the colonies remained [*PSI*^+^] after 8 hours of treatment. Surprisingly, parallel 5% DMSO with α -factor treated cultures showed 51% of the colonies were sectored at 4 hours, and 2.69% of the colonies were [*PSI*^+^] after 8 hours. These results suggest that DMSO-mediated curing is enhanced when the cell cycle is arrested, and are reminiscent of the large number of sectored colonies obtained by plating cells directly on 4% DMSO (Fig 1B). However, α -factor treatment alone had very little influence on curing (S2B Fig).

### DMSO treatment is associated with increased accumulation of soluble Sup35

**Fig 3:**
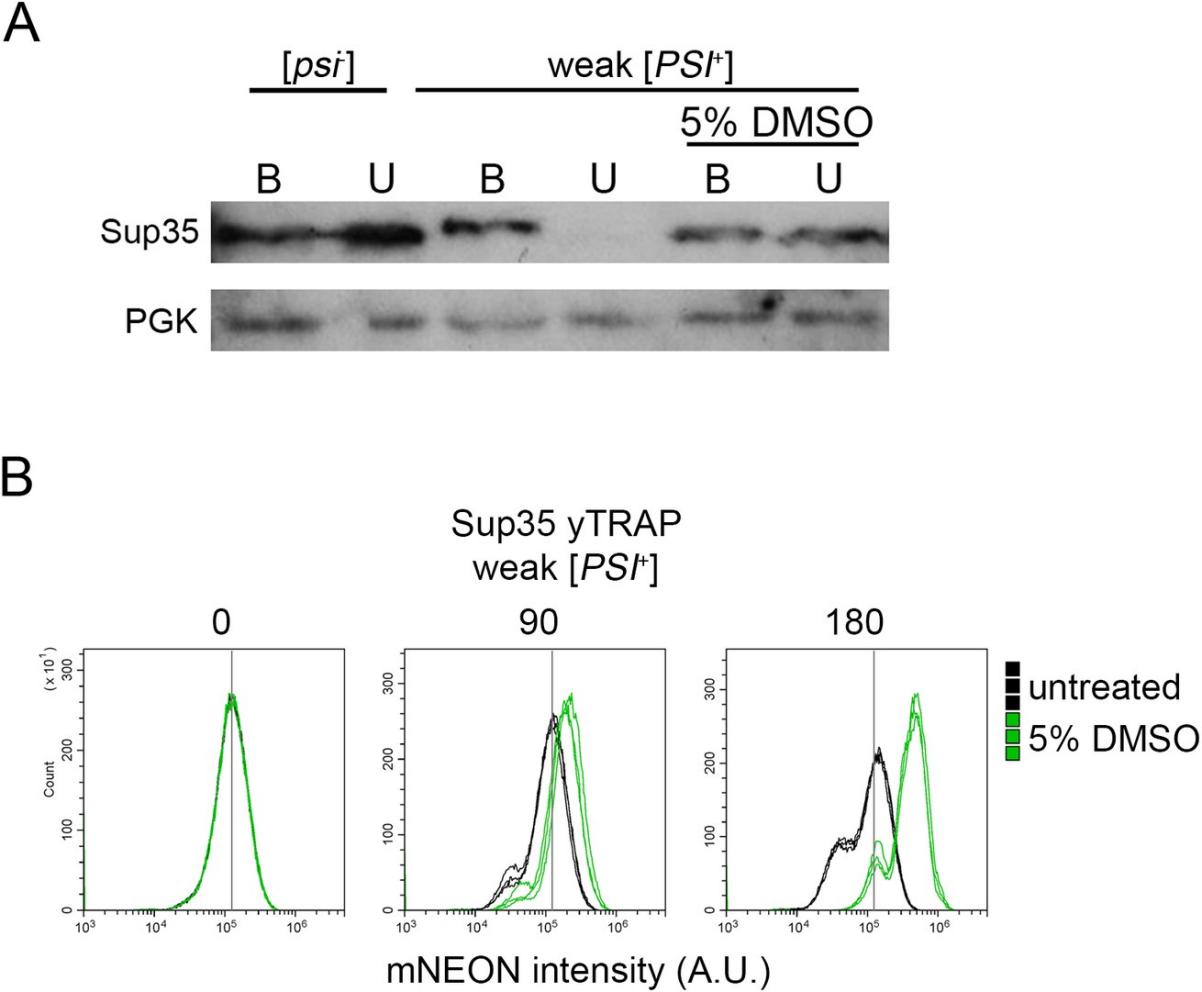
DMSO treatment is correlated with an increase in soluble Sup35. A. The indicated strains were untreated or treated with 5% DMSO for 24 hours and lysed. Lysates were boiled (B) or incubated at room temperature (U; unboiled) for 10 minutes prior to loading on SDS-PAGE. The indicated antibodies were used for Western blot analysis. Representative image is shown. B. Weak [*PSI*^+^] strains that contain an integrated version of the Sup35 yTrap construct (Newby et al., 2017) were grown in media alone (untreated) or supplemented with 5% DMSO (as indicated). Three independent cultures of each strain were subjected to flow cytometry at the indicated time points (above panels). The yTRAP signal is an output of the amount of soluble Sup35 available in each cell, as displayed by mNeonGreen intensity. Therefore, any shift to the right is an indication that there is more soluble Sup35 within the cell population than control samples. The fixed vertical line in all three panels provides reference relative to the baseline mNeonGreen peak observed in all samples at timepoint zero. 100,000 cells were counted per sample.

Weak [*PSI*^+^] cells treated with 5% DMSO for 24 hours results in [*psi*^-^] colonies (Fig 2A). However, it is unclear whether Sup35 is soluble immediately after treatment or whether the prion loss requires plating and cell division. Therefore, we ran Western blots of weak [*PSI*^+^] cultures that were treated with DMSO overnight. Under normal conditions, the Sup35 protein is soluble in [*psi*^-^] strains, but forms SDS-resistant aggregates in [*PSI*^+^] strains. Boiling [*PSI*^+^] lysates in the presence of SDS resolves Sup35 monomers by Western blot. As expected, unboiled lysates of [*psi*^-^] strains resolve Sup35 as a monomer, but [*PSI*^+^] strains do not. (Fig 3A). If weak [*PSI*^+^] strains require time after overnight DMSO treatment to give rise to [*psi*^-^] progeny, then we would expect that boiling lysates would be the only way to resolve Sup35 monomer. Our data shows that Sup35 is soluble after overnight 5% DMSO treatment, whether samples were boiled or unboiled (Fig 3A).

To determine whether soluble Sup35 is detectable upon short DMSO treatment, we utilized a recently developed tool called the yTRAP [yeast transcriptional reporting of aggregating proteins; 28]. The yTRAP system fuses an aggregation prone protein, such as Sup35, to a synthetic transcriptional activator (synTA). When Sup35 is soluble, the transcriptional activator binds to a synTA responsive promoter that drives the reporter gene, mNeonGreen. When Sup35 is aggregated, the fused transcriptional activator is no longer able to bind to the promoter and mNeonGreen is not expressed. Therefore, mNeonGreen intensity is a measure of the amount of soluble Sup35 within the cell. We integrated the Sup35 yTRAP into weak [*PSI*^+^] strains, and grew triplicate cultures to late log. Cultures were diluted to 0.2 optical density, and grown in media alone (untreated) or supplemented with 5% DMSO. At timepoint zero, all treated and untreated cultures gave similar yTRAP profiles (Fig 3B). 5% DMSO cultures exhibited a detectable increase in mNeonGreen intensity compared to untreated controls at 90 minutes, and a notable increase in mNeonGreen intensity by 180 minutes. It is possible that either more soluble Sup35 is generated over time, or an increasing proportion of [*psi*^-^] cells within the population contribute to the shift in yTRAP signal after 180 minutes. These shifts were maintained even after 360 minutes of treatment (S3A and S3C Fig). In contrast, yTRAP analysis of strong [*PSI*^+^] strains did not show clear shifts over time (S3B and S3C Fig), suggesting DMSO treatment of strong [*PSI*^+^] cells does not lead to a substantial amount of soluble Sup35 accumulation. These results correlate with the lack of strong [*PSI*^+^] curing we observed by our plating assays (Fig 1A and 1D).

### Hsp104 expression increases with DMSO treatment

**Fig 4:**
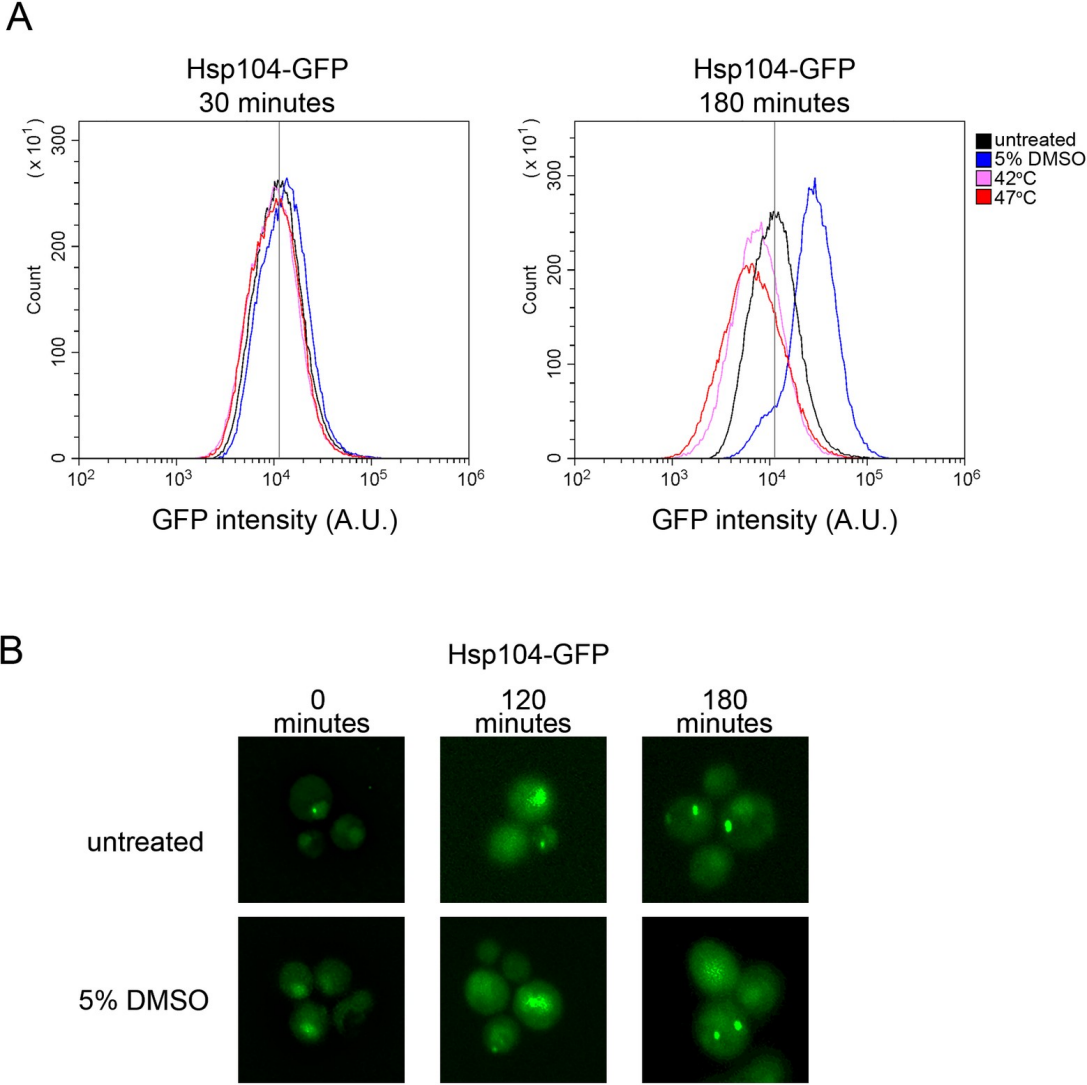
DMSO treatment increases Hsp104-GFP expression, but does not alter cytoplasmic fluorescence profiles. A. Strains containing an integrated Hsp104-GFP [20] were grown overnight, and then inoculated into media with starting optical densities of 0.2. Cultures were either untreated (black), grown at 42°C (pink), grown at 47°C (red), or in the presence of 5% DMSO (blue) for either 30 minutes (left panel) or 180 minutes (right panel). 100,000 cells were counted per strain by flow cytometry to assess the GFP fluorescent intensity per cell. Images are representative of three independent replicates (See S4 Fig). B. Strains from A were inoculated in to media alone (untreated) or 5% DMSO with starting optical densities of 0.1, and imaged at the indicated times. It should be noted that small intense puncta, and fluorescent dense regions were observed at all time points, in all triplicate trials, and in both untreated and 5% DMSO treated cells. Strains were imaged using 3D fluorescent microscopy. Each image is individually deconvolved to boost intensity and remove background, and therefore image intensities between panels cannot be compared. Images shown are maximum projections.

We asked whether DMSO treatment leads to changes in Hsp104 expression or changes in Hsp104 distribution within cells. 30 minute heat shock leads to marginal changes in Hsp104 expression, but is associated with asymmetric localization of Hsp104 in mother cells [20, 21]. Using a strain that contains Hsp104-GFP integrated into the *HSP104* locus and driven by the *HSP104* promoter [20], we treated log phase cultures with 5% DMSO for 30 minutes or 180 minutes. Cultures were subjected to flow cytometry analysis to determine whether GFP fluorescence increased in response to DMSO treatment. Given that Hsp104-GFP is the only source of fluorescence in these strains, the most plausible explanation for increased GFP fluorescence would be increased Hsp104 expression. 30 minutes of DMSO treatment resulted in cell populations with similar Hsp104-GFP fluorescence intensities compared to untreated controls (Fig 4A). Samples subjected to 30 minutes of heat shock also did not show any substantial increase in Hsp104-GFP fluorescence, which is consistent with previous reported results with the same strain [20]. After 180 minutes of treatment, only DMSO treated samples showed an increase in GFP intensity compared to parallel untreated cultures (Fig 4A). Additional trials showed similar results (S4A Fig). Since heat shock has been shown to increase Hsp104 expression, we expected that heat shock would similarly result in cell populations with higher GFP fluorescence intensity. The dramatic increase in the number of dead cells within the heat-treated populations, as detected by propidium iodide staining (S4 Fig), is consistent with cytotoxicity associated with heat shock [19]. This toxicity could explain the downshift in Hsp104-GFP fluorescence in heat treated samples (Fig 4). Our data suggest that DMSO treatment leads to increases in Hsp104 expression.

Short heat induced curing of weak [*PSI*^+^] is associated with asymmetric retention of Hsp104 in dividing cells [20, 21]. We asked whether DMSO treatment led to aggregation of Hsp104 and/or similar asymmetric detection. We found that Hsp104 cytoplasmic fluorescence was similar between untreated and 5% DMSO treated cells after 180 minutes. We did notice the presence of higher fluorescent intensity regions within the cell, and occasional small single puncta, but these features were found in both untreated and treated cultures at all time points. Microscopy data suggest that the mode of DMSO-mediated curing is independent of Hsp104 asymmetric retention (Fig 4B).

### The presence of the [PIN^+^] prion does not impact weak *[PSI*^*+*^*]* curing

**Fig 5:**
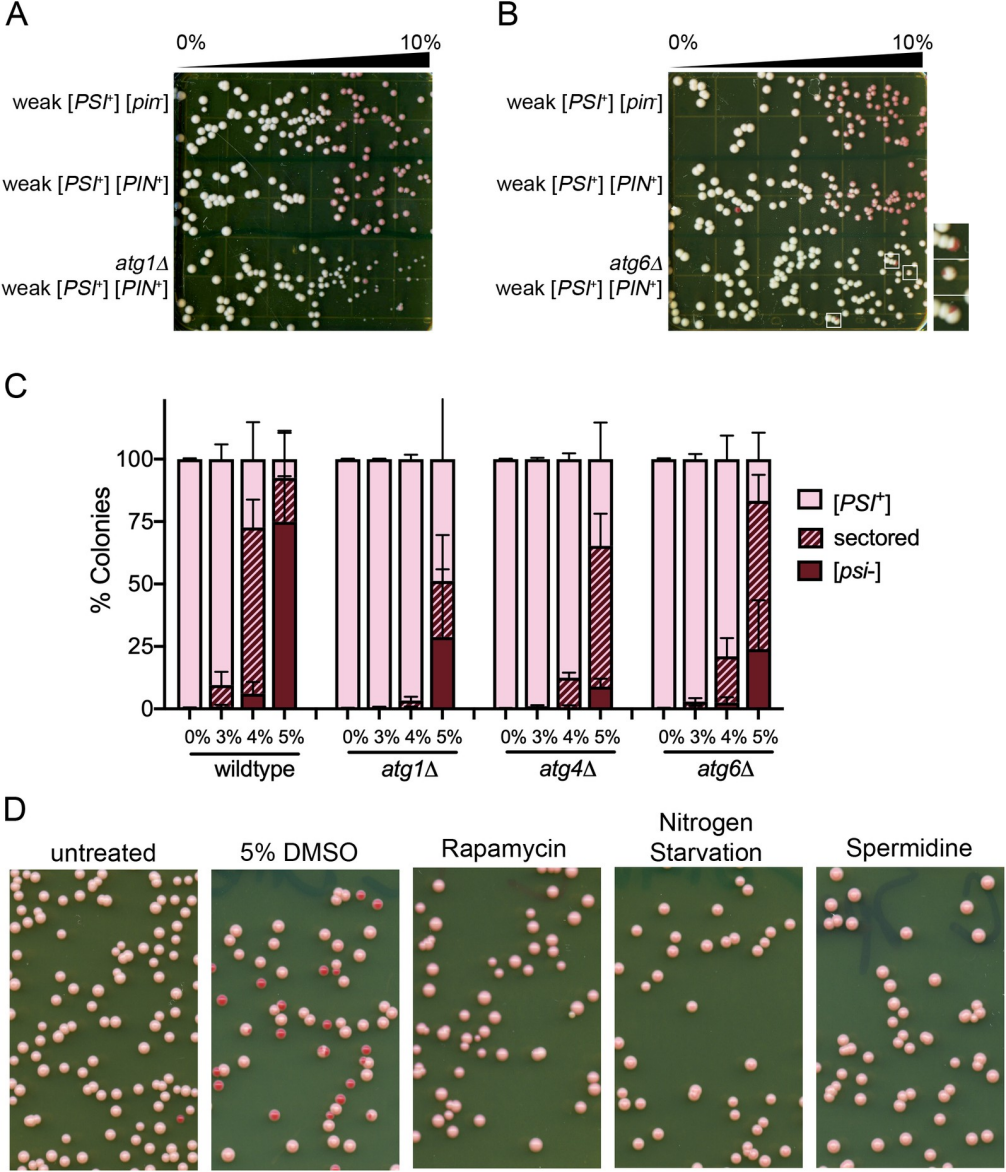
Autophagy mutants reduce DMSO-mediated curing, but autophagy alone is not sufficient to induce curing. A and B. The indicated strains were plated on 0%-10% DMSO gradient plates similar to Fig 1. Inset shows sectored colonies observed in *atg6Δ* weak [*PSI*^+^] strains. C. Triplicate independent cultures were grown in liquid culture overnight and plated on rich media containing the indicated percentage of DMSO. Over 1500 colonies were counted in three independent experiments. The percent of red colonies is plotted. Standard deviations are shown. The distributions of sectored and red colonies in *ATG* mutants in 4% and 5% DMSO treated samples are significantly different from wildtype strains with a *p-*value of <0.001 (chi-square test). D. Three independent cultures were grown in rich media overnight and inoculated into media alone (untreated), media containing 5% DMSO, media containing 0.2 ug/ml rapamycin, nitrogen starvation media, or media containing 4mM spermidine. Cultures were allowed to grow for three hours and then were plated on rich media. Plates are representative of 3 independent trials.

DMSO-mediated curing of weak [*PSI*^+^] does not change in the presence of another prion. The [*PIN*^+^] prion, also known as [*RNQ*^+^], is the misfolded form of the Rnq1 protein that has previously been shown to enhance de novo [*PSI*^+^] formation [29–31]. Weak [*PSI*^+^] [*PIN*^+^] strains showed similar curing to weak [*PSI*^+^][*pin*^-^] strains on gradient DMSO plates (Fig 5A), suggesting that [*PIN*^+^] does not interfere with DMSO-mediated curing of weak [*PSI*^+^].

### Autophagy related gene mutants reduce DMSO-mediated curing

DMSO treatment has been shown to induce autophagy in human hepatocytes [32]. To investigate the possible link between autophagy and DMSO-mediated prion loss in yeast, we selected an *ATG* (autophagy related gene) mutant that impacts macroautophagy, the process in which cytoplasmic material is compartmentalized into autophagosomes that are later fused to lysosomes for degradation. *ATG1* codes for a kinase involved in the initiation of macroautophagy. *atg1Δ* mutants are defective in autophagy, as well as exhibiting sporulation defects and decreased life span [33, 34] Using gradient plates, we found that *atg1Δ* mutants exhibited more toxicity at high DMSO concentrations compared to wildtype controls (Fig 5A). Despite the toxicity, *atg1Δ* colonies appeared to be more resistant to prion curing than wildtype cells throughout the gradient. These data indicate that absence of *ATG1* impairs prion curing by DMSO.

Next, we looked at a mutant that is involved in both macroautophagy and vacuolar sorting, *atg6Δ*. *ATG6* codes for a subunit of the phosphatidylinositol 3-kinase complex I and is an ortholog of mammalian Beclin protein. While toxicity observed in *atg6Δ* was similar to wildtype strains, very little curing was observed compared to wildtype controls (Fig 5B). Only a few sectored colonies were identified at higher concentrations (Fig 5B, inset).

To further investigate autophagy related gene mutants and DMSO-mediated curing, we tested curing at specific concentrations of DMSO. Using the number of sectored colonies as a way to detect subtle changes in curing, weak [*PSI*^+^] versions of wildtype, *atg1*Δ, and *atg6*Δ mutants were plated on 0%, 3%, 4% and 5% DMSO. (Fig 5C). While 66.6% of wildtype colonies were sectored at 4% DMSO, similar to Fig 1, autophagy related gene mutants displayed a different profile. Only a small percentage of colonies were sectored at 4% DMSO: *atg1Δ* mutants had 2.7%, and *atg6Δ* mutants had 18.5% sectored colonies.

We also looked at another autophagy related gene mutant, *atg4Δ*. *ATG4* codes for a cysteine protease that is involved in the processing of the Atg8 protein, which is required for the formation, elongation, and fusion of autophagic vesicles. Loss of *ATG4* is associated with decreased autophagy, as well as sporulation defects [35]. Compared to wildtype controls, *atg4Δ* had 11.9% sectored colonies at 4% DMSO (Fig 5C). Even at 5% DMSO, all autophagy mutants show a substantial population of colonies that were sectored or remained [*PSI*^*+*^] (Fig 5C).

The autophagy related gene mutants all exhibit autophagy defects. We wanted to know whether directly increasing autophagy could facilitate [*PSI*^+^] curing by itself. We treated cells with rapamycin or nitrogen starvation, both of which inhibit TOR signaling to induce autophagy [36], and spermidine, a polyamine that induces autophagy through acetyltransferase inhibition [37]. We did not observe curing of weak [*PSI*^+^] upon rapamycin treatment, nitrogen starvation, or spermidine treatment (Fig 5D). Only 5% DMSO treated strains showed curing of weak [*PSI*^+^].

## Discussion

Here, we show that the aprotic solvent, dimethyl sulfoxide (DMSO), leads to the rapid elimination of several [*PSI*^+^] prion variants (Fig 1). We used this treatment of curing to investigate novel quality control processes that are involved in prion elimination from cell populations. Our data suggest that DMSO increases Hsp104 expression (Fig 4), and curing can be dampened in autophagy related gene mutants (Fig 5). Taken together, DMSO possibly induces multiple processes, such as molecular chaperones and processes involving autophagy related genes, to cure cells of many different [*PSI*^+^] variants.

Guanidine hydrochloride has been found to cure many variants of [*PSI*^+^], as well as other prions such as [*PIN*^+^] and [*URE3*] [22, 30, 38, 39]. GuHCl cures both strong and weak variants of [*PSI*^+^] through the inactivation of Hsp104, resulting in propagon dilution with cell division [11, 14]. In contrast, while DMSO has no effect on strong [*PSI*^+^], DMSO cures variants that have a range of phenotypic colors between dark pink and light pink (Fig 1D). Furthermore, DMSO curing of weak [*PSI*^+^] is more rapid than GuHCl curing (Fig 2), suggesting that DMSO may facilitate curing by a process unrelated to Hsp104 inactivation. Compared to strong [*PSI*^+^], the weak [*PSI*^+^] variant has less Sup35 protein associated with prion aggregates, lower number of propagons, and the propagons tend to be larger [7, 17, 18]. Weak [*PSI*^+^] is also more unstable, resulting in 1 out of 1000 colonies being [*psi*^-^] [30]. It could be the low number of propagons as well as the size of the propagons that make the prion more susceptible to loss by DMSO.

Given distinctions between GuHCl and DMSO-mediated curing, and our observation that Hsp104 levels increase with DMSO treatment, as determined by GFP fluorescence intensity (Fig 4), it is worth considering what is known about [*PSI*^+^] curing by Hsp104 overexpression. Several models have been proposed, yet these models have very different modes of action. One model suggests that excess Hsp104 leads to the malpartition or asymmetric retention of prion aggregates in mother cells, eventually leading to prion loss after many cell division. In these studies, [*PSI*^+^] cells showed no Sup35 monomerization with Hsp104 overexpression [40]. Another model, which also results in no monomerization of Sup35, suggests that excess Hsp104 binds to specific regions of Sup35, preventing Hsp104-Hsp70-Hsp40 complexes from fragmenting prion filaments [41]. In contrast, it has also been proposed that excess Hsp104 leads to increased Sup35 solubilization by extracting, or trimming, monomers from the end of prion filaments [42]. Our data indicates that along with an increase in Hsp104 levels, soluble Sup35 levels also increase also after 180 minutes of treatment (Fig 3). While timing of these two observations suggest that Hsp104 expression and soluble Sup35 accumulation could be correlated, this increase in Sup35 monomer could be due to disassembly of the prion filament, accumulation of newly synthesized Sup35 protein that is unable to join pre-existing filaments, or newly obtained [*psi*^-^] cells in the population. Further experiments will be required to understand the link between increase in Hsp104 levels, Sup35 solubilization, and DMSO curing.

Since DMSO-mediated curing is associated with changes in Hsp104 expression, and is dampened by autophagy related gene mutants, it is possible that the mechanism of curing is not necessarily directly attached to one of these above models. Recently, it has been shown that a small portion of fluorescently decorated Sup35 aggregates in [*PSI*^+^] cells are recruited to the insoluble protein deposit (IPOD), a protein-rich membrane-less organelle adjacent to the vacuole that has been shown to be a site for amyloid deposition [43]. Authors show that prion aggregates are moved to IPOD by a myosin-based mechanism, and suggest that IPOD is a temporary storage site for excess aggregates until other quality control mechanisms are available to manage these aggregates. It is possible that the increase in Hsp104 expression, the proximity of prion aggregates in IPOD, and autophagy associated mechanisms together result in rapid curing of the prion during DMSO exposure.

In human hepatocytes, DMSO treatment enhances autophagy induction, detected through the proteolytic processing of the human ATG8 homolog, LC3 [32]. DMSO also reduces aggregation associated with exon I of the Human Huntingtin gene (HTT) in tissue culture. In this study, we observed that DMSO-mediated curing is dampened in the presence of *atg* deletion mutants, but still occurs relative to non-DMSO treated cultures. Additionally, treatment of [*PSI*^+^] cultures with Rapamycin, N-starvation, or Spermidine, each of which have been shown to induce autophagy [36,37], does not promote [*PSI*^+^] curing. These data suggest that autophagy is neither necessary nor sufficient for [*PSI*^+^] curing through DMSO, but the presence of functional *ATG1*, *ATG4* and *ATG6* proteins may play some as yet undetermined role in improving DMSO-mediated curing. Further studies focused on how changes to multiple protein quality control systems influence prion loss will provide important insight into understanding how these pathways work together to impact protein aggregation associated with human diseases.

## Materials and methods

### Yeast strains and growth conditions

[*psi*^-^] [strain L2910; 4], weak [*PSI*^+^] [L1759; 14] and strong [*PSI*^+^] [L1763; 14] strains are derivatives of 74-D694 (*Mat****a*** *ade1-14 leu2-3*,*112 his3-Δ200 trp1-289 ura3-52*), and *hsp104* deletion strains in the 74-D694 background were disrupted with a *LEU2* cassette [L1804; 44]. These strains were kind gifts from Susan W. Liebman. The 74-D694 strain integrated Hsp104-GFP was a kind gift from Tricia Serio [D170; 20]. Disruptions of *atg1Δ* (M336, M337), *atg4Δ* (M340, M341), and *atg6Δ* (M343, M344) were engineered in this study are described below. The Sup35 yTRAP integrating plasmid [pGAN200; Sup35NM inserted into ccdB site of the yTRAP plasmid; 28], was a kind gift from Ahmed Khalil and Susan Lindquist. Plasmids were linearized and integrated into [*psi*^-^] (L2910), weak [*PSI*^+^] (L1759) and strong [*PSI*^+^] (L1763). Strains used in this study are indicated in Table 1.

**Table 1:** Strains used in this study.

Strain number	Genotype	Reference
L2910	*Mat****a*** *ade1-14 leu2-3*,*112 his3-Δ200 trp1-289 ura3-52* [*psi*^-^]	[4]
L1759	*Mat****a*** *ade1-14 leu2-3*,*112 his3-Δ200 trp1-289 ura3-52* weak [*PSI*^+^]	[14]
L1763	*Mat****a*** *ade1-14 leu2-3*,*112 his3-Δ200 trp1-289 ura3-52* strong [*PSI*^+^]	[14]
L1804	*Mat****a*** *ade1-14 leu2-3*,*112 his3-Δ200 trp1-289 ura3-52 hsp104*::*LEU2*	[44]
D170	*Mat****a*** *ade1-14 leu2-3*,*112 his3-Δ200 trp1-289 ura3-52 HSP104-GFP*:*KANMX6*	[20]
M517	*Mat****a*** *ade1-14 leu2-3*,*112 his3-Δ200 trp1-289 ura3-52* weak [*PSI*^+^] *Sup35-SynTA*, *pSynTA-mNeonGreen (yTRAP)*	This study
M518	*Mat****a*** *ade1-14 leu2-3*,*112 his3-Δ200 trp1-289 ura3-52* strong [*PSI*^+^] *Sup35-SynTA*, *pSynTA-mNeonGreen (yTRAP)*	This study
M336, M337	*Mat****a*** *ade1-14 leu2-3*,*112 his3-Δ200 trp1-289 ura3-52 atg1*::*HIS3*	This study
M340, M341	*Mat****a*** *ade1-14 leu2-3*,*112 his3-Δ200 trp1-289 ura3-52 atg4*::*HIS3*	This study
M343, M344	*Mat****a*** *ade1-14 leu2-3*,*112 his3-Δ200 trp1-289 ura3-52 atg6*::*HIS3*	This study

All strains were grown at 30°C using standard media and cultivation procedures (Sherman, Fink, and Hicks, 1986), except when mentioned. Gradient plates were made by pouring 20 mls of 10% DMSO YPD agar mixture into plates held at a tilted angle on four paper towels. After solidification, 20 mls of YPD agar was poured on level plates. Plates were allowed to equilibrate for 12–24 hours before plating cells. Plates and liquid cultures were made to contain a final concentration of the indicated DMSO percentages, or other treatments such as GuHCl, rapamycin, or spermidine. Starvation liquid media contained only yeast nitrogen base and glucose [45, 46]. After incubation in liquid media, cultures were plated onto rich media and allowed to grow for single colonies.

### *[PSI*^*+*^*]* color assay

All strains used in this study contained the *ade1-14* allele, which contains an *ochre* nonsense mutation within the *ADE1* open reading frame. The Sup35 translation termination factor is soluble is [*psi*^*-*^] cells and is available for stopping translation, resulting in a truncated non-functional Ade1 protein and accumulation of red pigment. In [*PSI*^+^] cells, less Sup35 protein is available for termination leading to readthrough of some *ade1-14* transcripts, resulting in white colony color. Strong [*PSI*^+^] has little soluble Sup35 protein and appears white compared to weak [*PSI*^+^] colonies, which has more soluble Sup35 protein and appears pink.

### Autophagy related gene disruptions

Homologous recombination was used to replace *ATG* genes with a *HIS3* gene in 74-D694 [*PIN*^+^] strains, as previously described in Manogaran et al. [47]. Briefly, PCR product containing flanking 5’ and 3’ ends of specific *ATG* genes and the *HIS3* open reading frame were transformed into strains. His+ transformants were confirmed for gene replacement by PCR and strains were checked for the [*PIN*^+^] prion by the presence of Rnq1-GFP aggregates. Weak [*PSI*^+^] was cytoduced into mutant strains using *kar1* donor strain (D132; *Mata SUQ5 ade2-1 kar1 lys1-1 his3-11*,*15 leu1 cyh*^*R*^ *weak* [*PSI*^+^]). Cytoductants were confirmed by change in colony color, the presence of Sup35-GFP aggregates, and curing on GuHCl.

### Biochemical analysis of yeast lysates

Cell lysates from indicated strains were prepared according to Sharma et al. [26]. Approximately 100 μg of crude lysate was treated with 2% SDS sample buffer in the presence of 8% betamercaptoethanol. Samples were either boiled or left at room temperature for 10 minutes prior to loading. Samples were run on 10% SDS-PAGE gels and subjected to standard Western blot procedures using anti-Sup35C antibody, anti-PGK antibody (Life Technologies), and secondary HRP antibody.

### Flow cytometry

Sup35 yTRAP strains or Hsp104-GFP strains were grown to late log. Strains were inoculated in fresh media to an optical density of 0.1–0.2 and grown for the indicated times. Flow cytometry was performed using a Cytoflex Flow Cytometer (Beckman Coulter) using a 488 nm laser and a FITC-A filter to measure GFP fluorescence intensity in single cells. Propidium iodide staining was used to detect dead cells upon DMSO and heat treatment (see S4 Fig). 100,000 cells were counted per sample. Histograms were generated using CytExpert Software.

## Supporting information

S1 Raw images (TIF)

S1 Fig Red colonies remain red after restreaking, but many pink colonies are unstable. Red, sectored, and pink colonies from DMSO treatment (Fig 1B) were streaked on rich media to assess whether the prion was either maintained or lost within the population. A. Red, pink, or sectored colonies obtained from DMSO treatment (color of source colony) were restreaked on rich media. Shown is a representative plate in in which red source colonies give rise to red colonies upon restreaking, but pink and sectored source colonies give rise to both red and pink colonies. B. Red, sectored, and pink source colonies (X-axis) were restreaked and assessed for the resulting colony color. The streaks that gave back populations that were completely red, a combination of pink and red, or only pink are indicated. Ten colonies from each category were tested.
(TIF)

S2 Fig α-factor treatment in the presence and absence of DMSO. A. Mean optical density readings triplicate cultures shown in Fig 2D treated with 5% DMSO in the presence (orange) or absence (gray) of 50 μM α -factor. B. The percent of colonies that were [*PSI*^+^] (left panel) and optical densities of cultures (right panel; starting OD_600_ was 0.007) in the presence (orange) or absence (gray) of 50 μM α -factor. Each point represents approximately 500 colonies per trial (in triplicate). All data represents means; error bars represent standard deviations.
(TIF)

S3 Fig DMSO treated weak [*PSI*^+^] strains show a shift in yTRAP signal, but strong [*PSI*^+^] strains do not. A. Flow cytometry analysis of cells containing the Sup35 yTRAP. The yTRAP assay for samples in Fig 3A are shown for timepoints between 0 and 360 minutes. The fixed vertical line in all three panels provides reference relative to the baseline mNeonGreen peak observed in all samples at timepoint zero. 100,000 cells were counted per sample. B. Triplicate cultures containing strong [*PSI*^+^] that contain an integrated version of the Sup35 yTrap construct (Newby et al., 2017) were grown in media alone (untreated) or supplemented with 5% DMSO (as indicated).
C. One culture (trial 1) of weak [*PSI*^+^] (left panel) and strong [*PSI*^+^] (right panel) followed over time. 100,000 cells were counted per sample.
(TIF)

S4 Fig DMSO treatment increases Hsp104 expression in all trials, but the decreased fluorescence in heat treated cells is correlated with enhanced cell death. A. Three separate cultures were grown overnight and inoculated into media alone or 5% DMSO. Left panel shows three trials of untreated and 5% DMSO treated samples. Right panel shows untreated samples compared to samples grown at 42°C and 47°C. Bottom panel shows that percentage of cells that were dead after the indicated treatments, as assayed by propidium iodide.
(TIF)
