# Supplementary figures and images for: DMSO-mediated curing of several yeast prion variants involves Hsp104 expression and protein solubilization, and is decreased in several autophagy related gene (atg) mutants
Source: PLoS One. 2020 Mar 5;15(3):e0229796. doi: 10.1371/journal.pone.0229796 (PMC7058316; doi:10.1371/journal.pone.0229796)

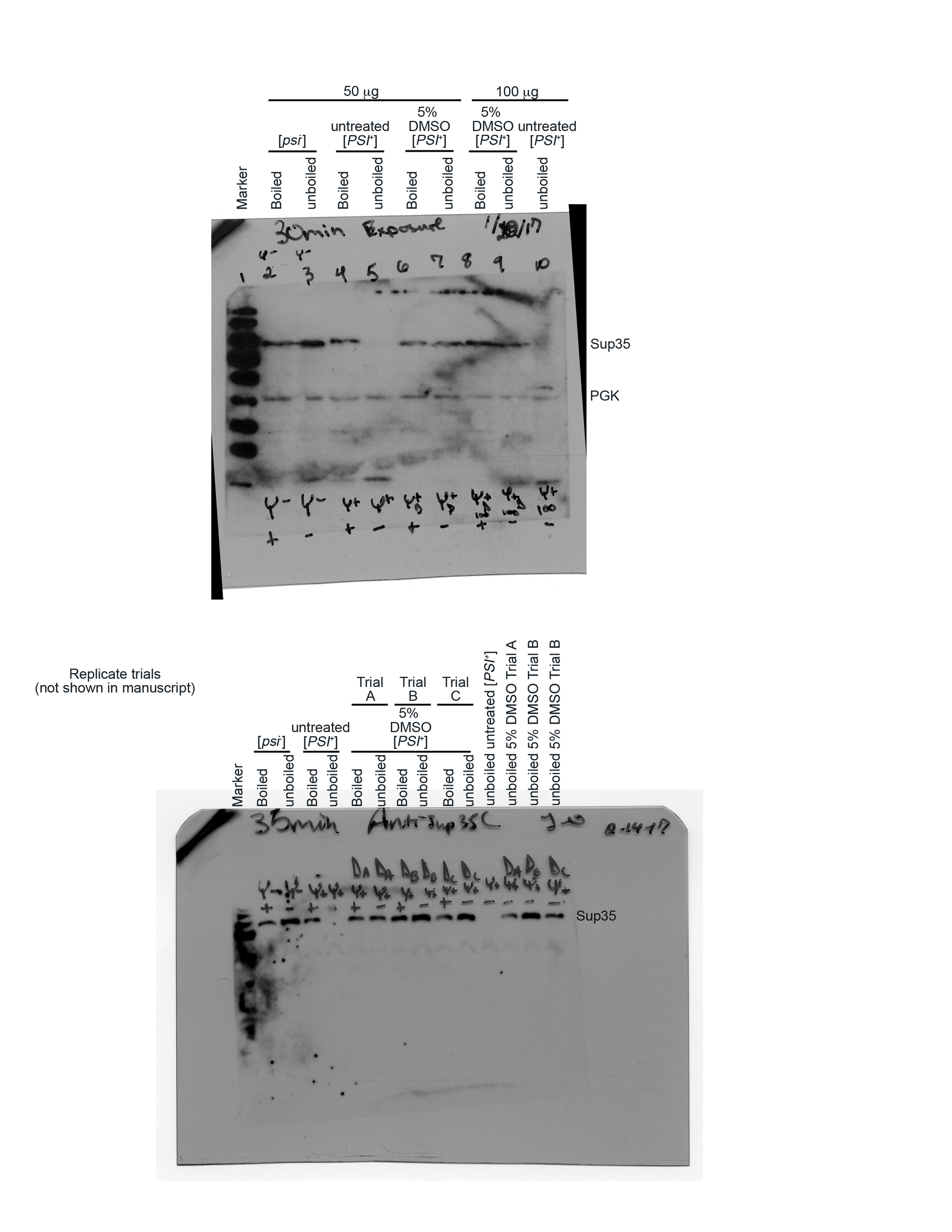

Supplement: S1 Raw images — (TIF) [file pone.0229796.s001.tif]

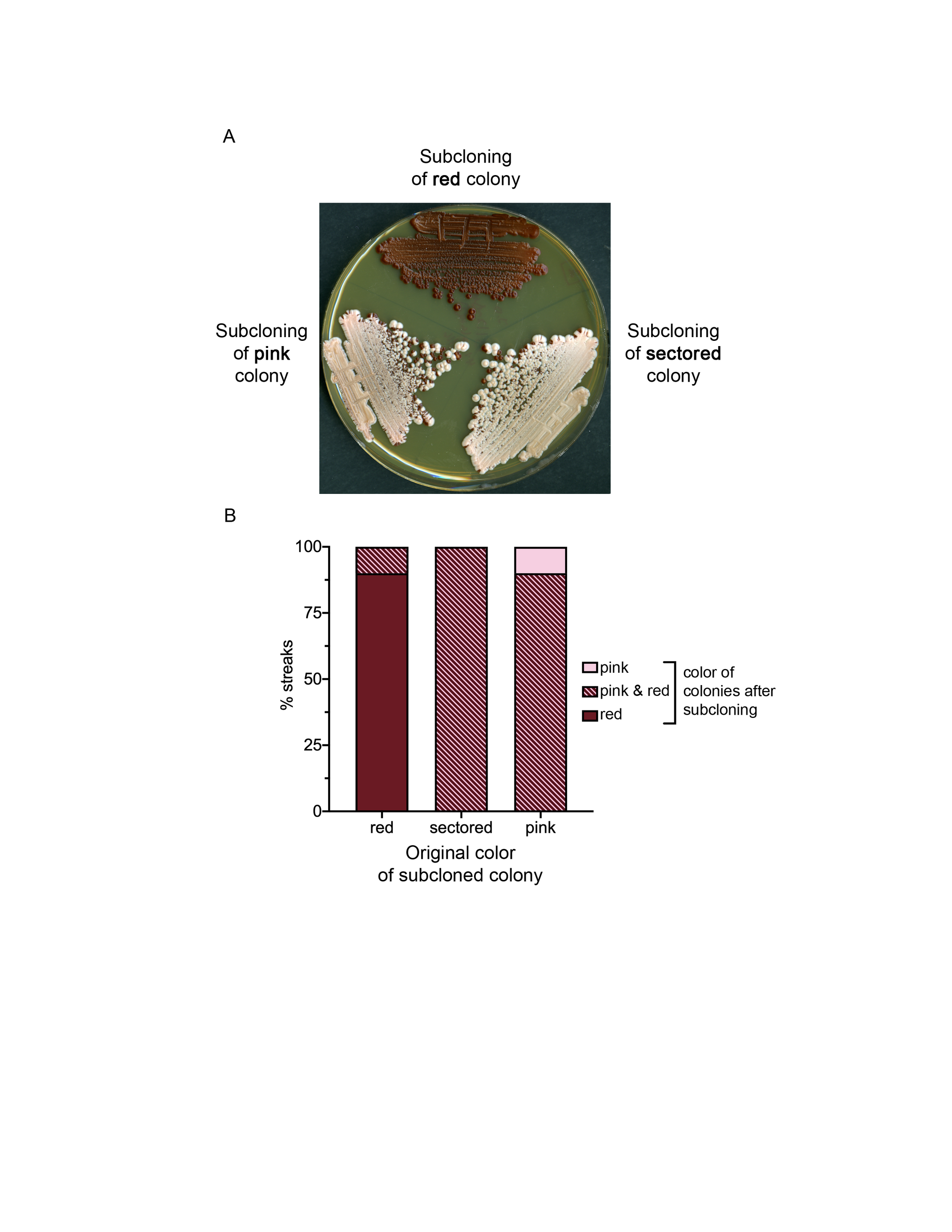

Supplement: S1 Fig — Red, sectored, and pink colonies from DMSO treatment (Fig 1B) were streaked on rich media to assess whether the prion was either maintained or lost within the population. A. Red, pink, or sectored colonies obtained from DMSO treatment (color of source colony) were restreaked on rich media. Shown is a representative plate in in which red source colonies give rise to red colonies upon restreaking, but pink and sectored source colonies give rise to both red and pink colonies. B. Red, sectored, and pink source colonies (X-axis) were restreaked and assessed for the resulting colony color. The streaks that gave back populations that were completely red, a combination of pink and red, or only pink are indicated. Ten colonies from each category were tested. (TIF) [file pone.0229796.s002.tif]

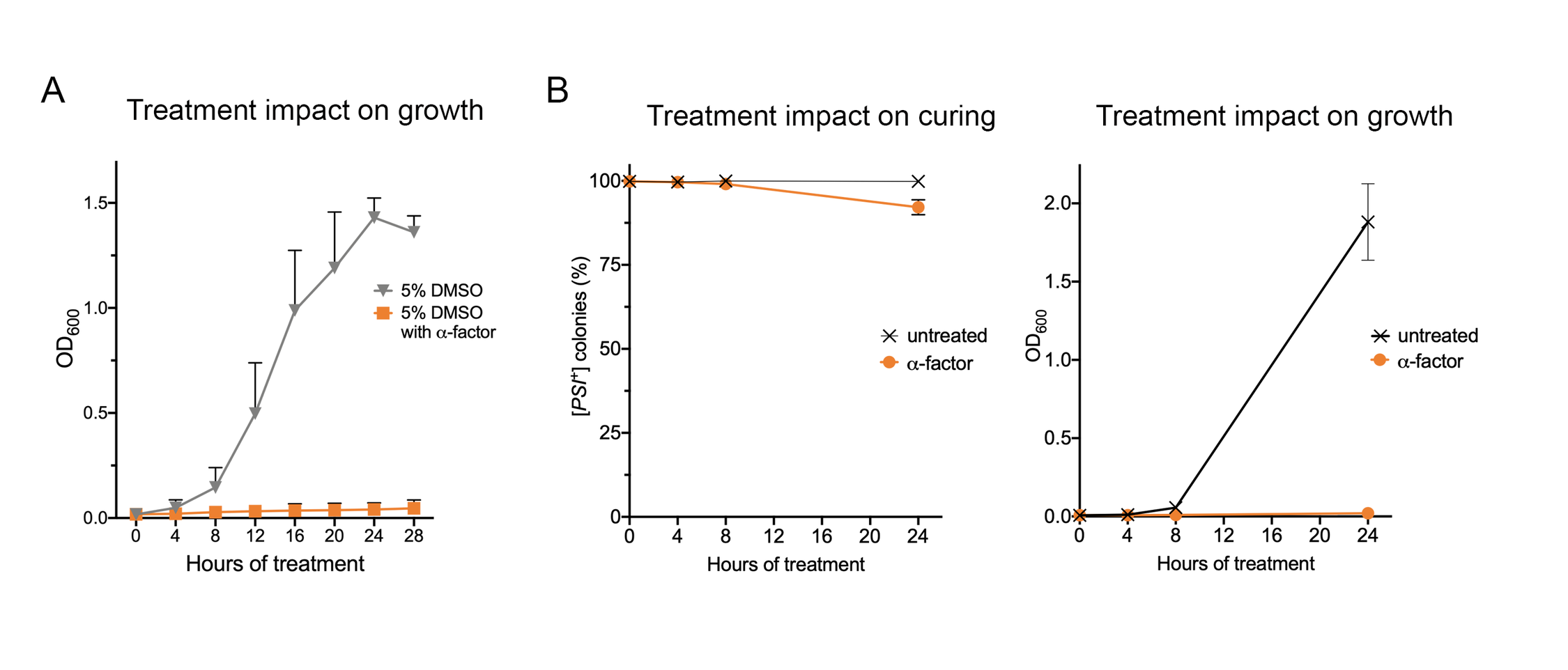

Supplement: S2 Fig — A. Mean optical density readings triplicate cultures shown in Fig 2D treated with 5% DMSO in the presence (orange) or absence (gray) of 50 μM α -factor. B. The percent of colonies that were [PSI+] (left panel) and optical densities of cultures (right panel; starting OD600 was 0.007) in the presence (orange) or absence (gray) of 50 μM α -factor. Each point represents approximately 500 colonies per trial (in triplicate). All data represents means; error bars represent standard deviations. (TIF) [file pone.0229796.s003.tif]

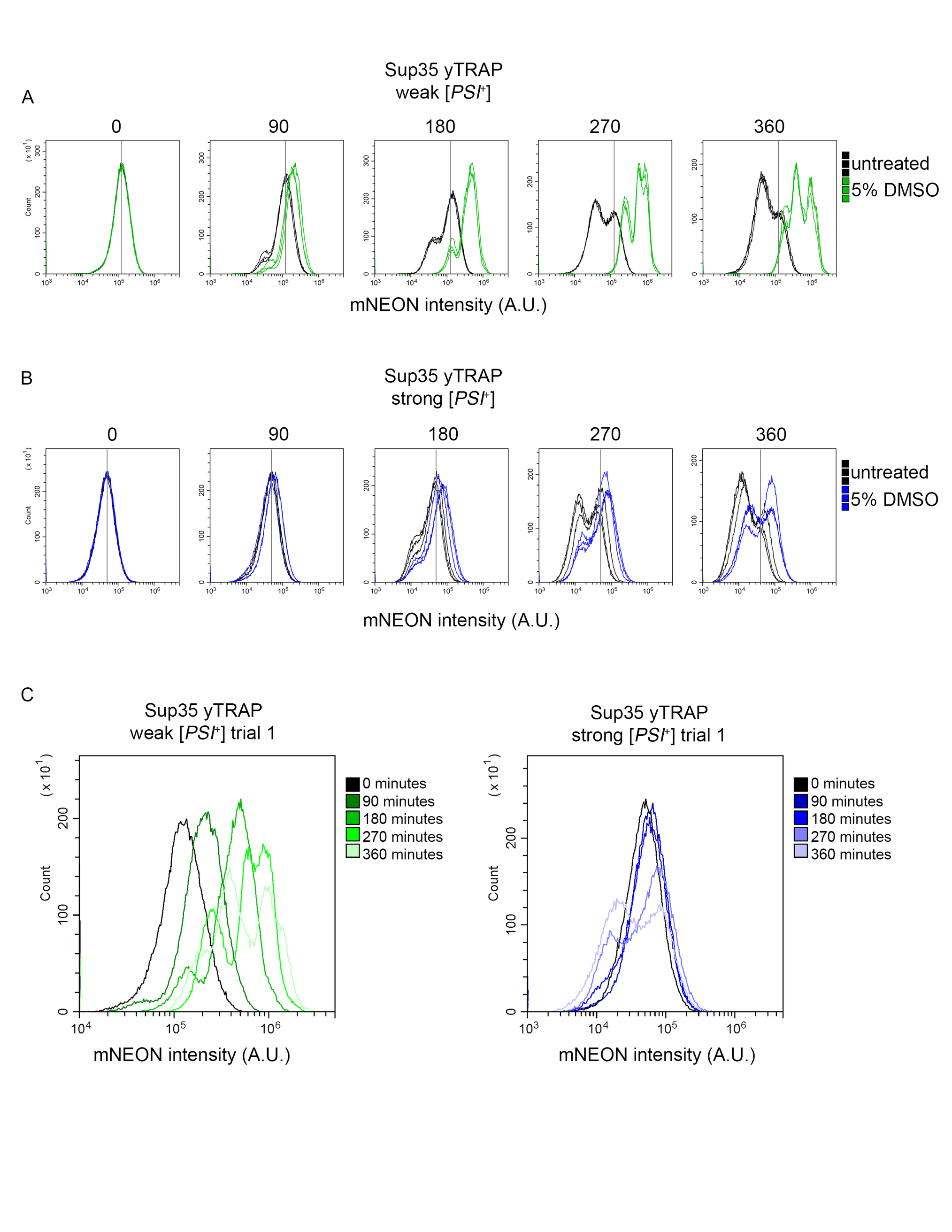

Supplement: S3 Fig — A. Flow cytometry analysis of cells containing the Sup35 yTRAP. The yTRAP assay for samples in Fig 3A are shown for timepoints between 0 and 360 minutes. The fixed vertical line in all three panels provides reference relative to the baseline mNeonGreen peak observed in all samples at timepoint zero. 100,000 cells were counted per sample. B. Triplicate cultures containing strong [PSI+] that contain an integrated version of the Sup35 yTrap construct (Newby et al., 2017) were grown in media alone (untreated) or supplemented with 5% DMSO (as indicated). C. One culture (trial 1) of weak [PSI+] (left panel) and strong [PSI+] (right panel) followed over time. 100,000 cells were counted per sample. (TIF) [file pone.0229796.s004.tif]

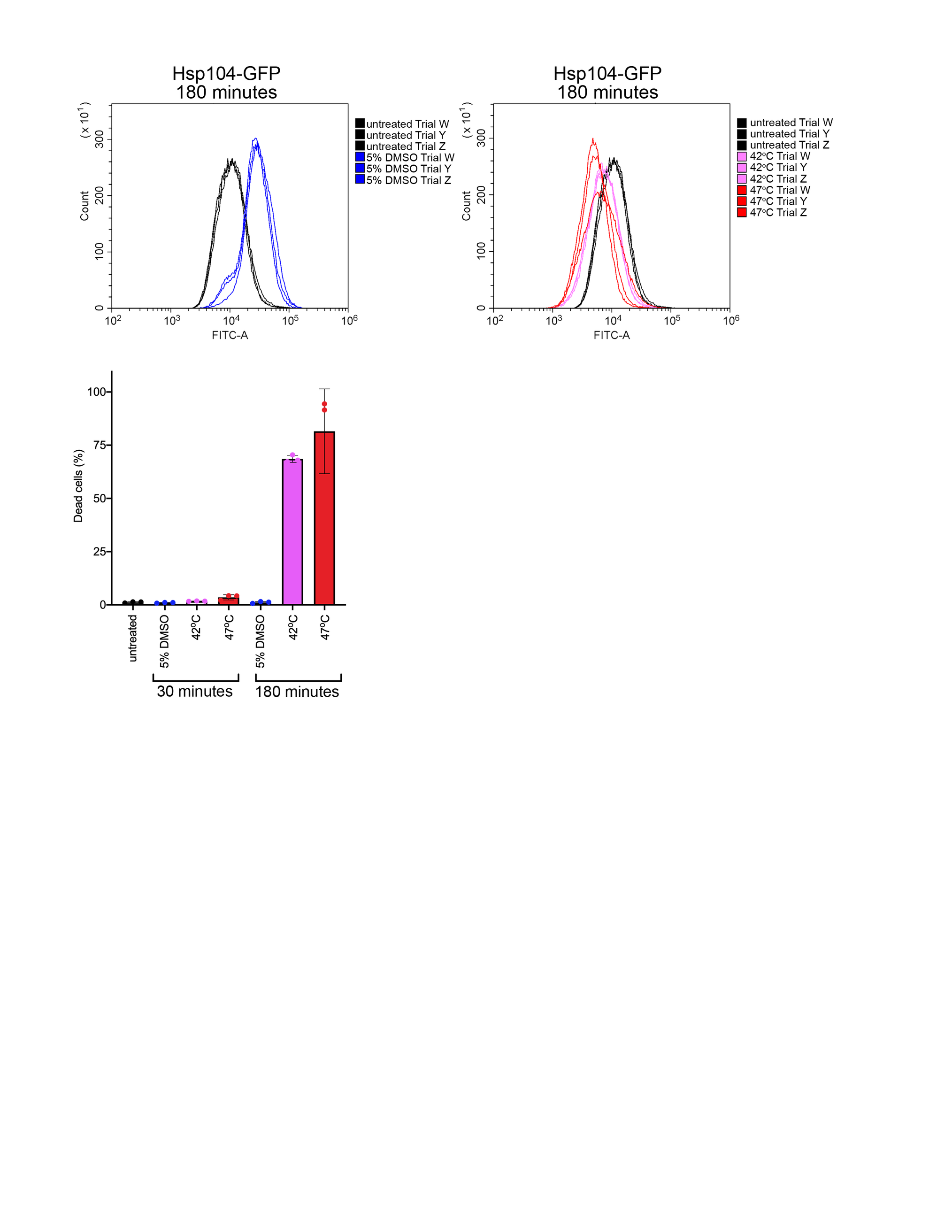

Supplement: S4 Fig — A. Three separate cultures were grown overnight and inoculated into media alone or 5% DMSO. Left panel shows three trials of untreated and 5% DMSO treated samples. Right panel shows untreated samples compared to samples grown at 42°C and 47°C. Bottom panel shows that percentage of cells that were dead after the indicated treatments, as assayed by propidium iodide. (TIF) [file pone.0229796.s005.tif]
